# Polymer Thermophoresis by Mesoscale Simulations

**DOI:** 10.1021/acs.macromol.4c01656

**Published:** 2024-12-04

**Authors:** Lisa Sappl, Christos N. Likos, Andreas Zöttl

**Affiliations:** Faculty of Physics, University of Vienna, Boltzmanngasse 5, 1090 Vienna, Austria

## Abstract

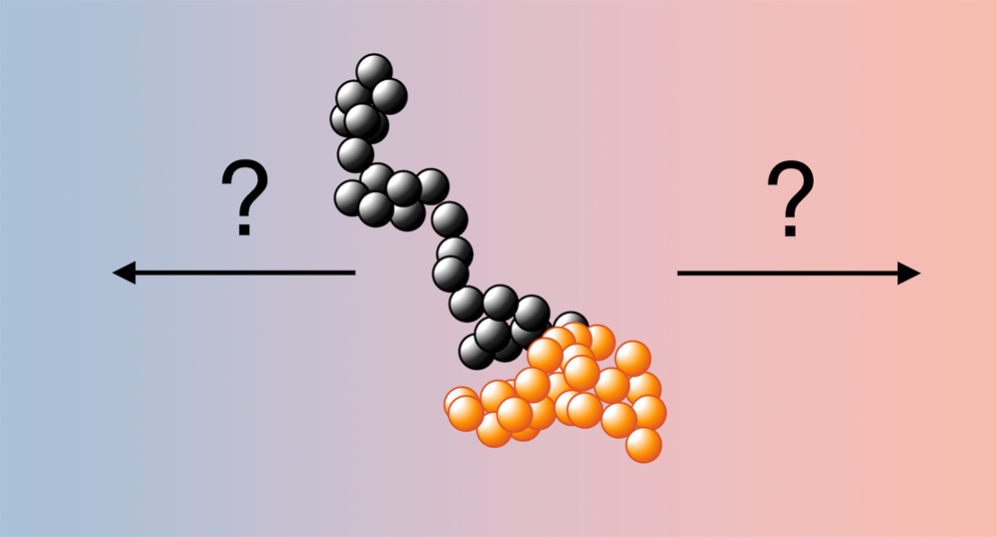

We employ mesoscopic simulations to study the thermophoretic motion of polymers in a solvent via multiparticle collision dynamics (MPCD). As the usual solvent–monomer collision rules employed in MPCD involving polymers fail to cause thermophoresis, we extend the technique by introducing explicit solvent–monomer interactions, while the solvent molecules remain ideal with respect to one another. We find that with purely repulsive polymer–solvent interaction, the polymer exhibits thermophilic behavior, whereas to display thermophobic behavior, the polymer–solvent potential requires the presence of attractions between solvent particles and monomers, in accordance with previous experimental findings. In addition, we observe that the thermophoretic mobility is independent of polymer length in the observed regime, again in agreement with experiments. Finally, we investigate the thermophoretic behavior of block copolymers, demonstrating that the thermophoretic mobility can be obtained by linear interpolation, weighted by the relative lengths of the two blocks.

## 1 Introduction

Thermophoresis is known as the directed motion of colloids, polymers, and molecules in a solution with applied temperature gradient. Similar as diffusiophoresis (directed motion in a concentration gradient), the thermophoretic motion of particles is force-free and it is relatively weak compared to particles which are directly subjected to external forces. For sufficiently small temperature gradients **∇***T*, the so-called thermophoretic velocity, i.e. the average velocity of particles in a temperature gradient, is linearly related to **∇***T*
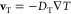
1through the thermophoretic mobility (or thermodiffusion coefficient) *D*_T_, which can be determined experimentally or in simulations, and depends on particle and solvent properties.^1,2^ Notably, it had been demonstrated that **v**_T_ can point against (*D*_T_ > 0) or with (*D*_T_ < 0) the direction of the temperature gradient, and respectively the particle moves either toward the cold side and is therefore thermophobic, or to the hot side and it is hence thermophilic. The ratio of the thermodiffusion coefficient *D*_T_ to the particle’s diffusion coefficient *D* is called Soret coefficient *S*_T_ = *D*_T_/*D* and has physical dimension of inverse temperature. It has been determined for many colloidal, polymeric, and molecular systems, and it had been demonstrated that most particles are thermophobic, while some are thermophilic.

The thermophoretic effect was first observed by Ludwig^3^ and later systematically investigated in saline solutions by Soret.^4^ Early thermal field-flow fractionation experiments, utilizing the thermophoretic effect, showed to separate polymers in solution,^5^ and it was found that that the thermophoretic mobility is independent of the degree of polymerization.^6^ Schimpf and Giddings also found that in diblock copolymers, the thermophoretic mobility is dominated by the well-soluble block due to the conformations that these copolymers assume, in which the poorly soluble block forms the compact core of the molecule and is shielded by the well soluble block that forms the outer layer.^7^ More recent experimental methods involve optical detection methods and optical (laser) temperature control. It could be shown that the independence of the thermophoretic mobility on the degree of polymerization for polymers only upholds as long as the polymer length exceeds the Kuhn length.^8,9^ For systems with identical chemistry of solvent and solute, in the limit of small solvent molar mass in relation to solute molar mass, the thermophoretic mobility reaches a universal value that is only dependent on solvent viscosity η.^10^ Experiments on poly(ethylene oxide) (PEO) resulted in a sign change of the thermophoretic mobility when modifying solvent composition^11^ or temperature.^12^ Kita et al. also observed a thermally induced sign change using a different polymer.^13^ We note that the thermophoretic effect might have played a role in prebiotic evolution, demonstrated by the fact that a temperature gradient allows for accumulation of vital substances in the origin of life.^14−16^

Experiments with charged colloids and polymers have established the crucial role played by thermal fluctuations and by the interplay between thermophoresis and body forces excerted on the nanobeads or the DNA by electric fields.^17−19^ In particular, the proportionality relation between thermophoretic velocity and temperature gradient shown in eq 1 has been found to be valid only at small Peclet numbers *Pe* for the case of charged molecules,^17^ where also thermal fluctuations are important and local thermodynamic equilibrium holds.^19^ In this work, *Pe* < 1, strong thermal fluctuations are present and there are no electric charges and no body-forces acting on the polymers.

Computer simulations using nonequilibrium molecular dynamics (NEMD) showed that *n*-alkane binary mixtures always reach a steady state in which the heavier component accumulates in the cold region, whereas the lighter component is more concentrated in the hot region,^20^ in agreement with experiments. Computer simulations have also been used to study thermodiffusion in liquid binary alloys^21^ and in colloidal suspensions.^22,23^ Simulations using full molecular dynamics for both, polymer and solvent, showed a strong correlation between solvent quality and Soret coefficient.^24^ Despite the large amount of research on the thermophoretic effect, the thermophoretic mobility is still unpredictable for most systems, both in magnitude and sign.

Numerical modeling of colloidal and polymeric thermophoresis is challenging due to the complexity and relevance of solute–solvent interactions as well as capturing long-range hydrodynamic flows of the force-free motion of colloids or polymers. To overcome different length and time scales, as well as including hydrodynamic flow fields and thermal motion, coarse-grained methods such as multiparticle collision dynamics (MPCD) or dissipative particle dynamics (DPD) are well-established simulation techniques. We will use MPCD which had been successfully applied to study the dynamics of colloidal suspensions and polymer solutions out-of-equilibrium.^25^ It had been used to investigate polymers under shear flow (see, e.g. ref (26–29)), and under the influence of external forces.^30^ So far, MPCD had been applied to the thermophoresis of thermophobic and thermophilic colloids.^22,23^ It had been demonstrated that the specific colloid–solvent interaction in the presence of a temperature gradient determines thermophoretic behavior.^22^ Here we propose a new method to simulate the thermophoresis of homopolymers as well as block copolymers using the MPCD method. Our work demonstrates that the specific monomer–solvent interaction plays a major role in determining both magnitude and sign of the thermophoretic mobility. Our simulation results match well with experimental results by de Gans et al.,^11^ as we can reproduce both a sign change of *D*_T_ induced by changing the solute–solvent interactions, and the decoupling of solvent quality and thermophoretic motion.

The work is organized as follows. In Chapter 2, we introduce the methods we used for our simulations. In Chapter 3, we present equilibrium properties of our polymer system. The following Chapter 4 demonstrates the results for polymers in a temperature gradient. At last, in Chapter 5, we draw our conclusions and present the reader with an outlook.

## 2 Methods

### 2.1 MPCD Solvent Featuring a Temperature Gradient

The solvent is modeled on a coarse-grained level represented by *n*_s_ effective point particles, each with mass *m*. As the solvent particles are modeled as ideal, the multiparticle collision dynamics (MPCD) algorithm propagates the particles using two alternating steps: ballistic movement, and collision.^31^ During the ballistic movement, the new position **r**_*i*_(*t* + *h*) of particle *i*, *i* = 1, ..., *n*_s_ is calculated using its old position **r**_*i*_(*t*) and velocity **v**_*i*_(*t*), as well as time step size *h*, i.e.
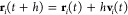
2For the collision step, first, space is divided into cubic cells of side length *a*. Within each of these cells, first the relative velocities **w**_*i*_(*t*) of the particles with respect to the center of mass velocity in the cell **v**_cm_(*t*) are calculated, i.e.
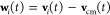
3where
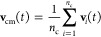
4Here, the quantity *n*_c_ stands for the number of particles in the respective cell, which is fluctuating, i.e., in the MPCD only the average number of particles per cell, ⟨*n*_c_⟩, is fixed. Then, to mimic a collision, the relative velocities are rotated by a fixed angle α about a randomly chosen unit vector , and added back to the center of mass velocity to calculate the new velocities
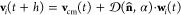
5The matrix  is the standard universal rotation matrix defined by its axis  and angle α. To ensure Galilean invariance, the placement of collision cells has to be shifted by a random vector with uniformly distributed independent components ∼*U*(−*a*/2, *a*/2) for each time step.

**Figure 1:**
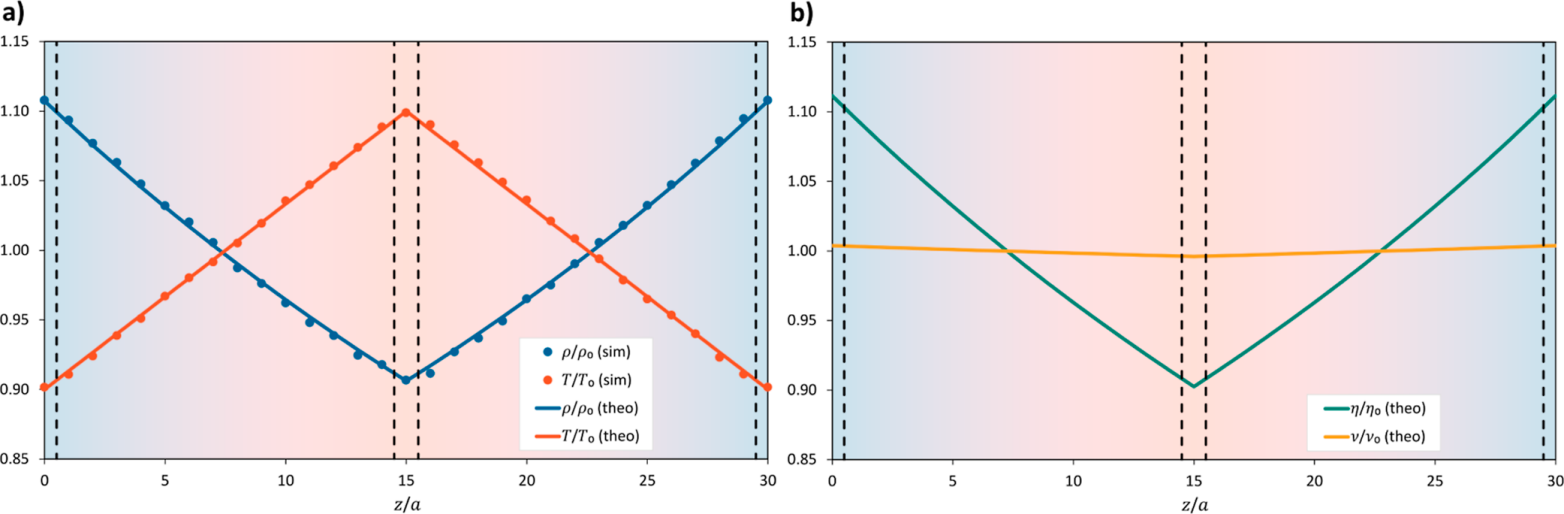
(a) The background solvent temperature (red) and density (blue) profiles along the *z*-axis with the employed thermostatting method of a cold slab at *z* = 0 and a hot slab at *z* = *l* (here *l* = 15*a*) using periodic boundary conditions. The continuous lines stand for the theoretical profiles following eqs 10 and 11, while dots represent simulation results (as slab and time averages) using 50 independent realizations. (b) Shows the theoretical local dynamic viscosity (green) and kinematic viscosity (yellow) as calculated in eqs 12–15. We use here the reference viscosities η_0_ = η(*T* = *T*_0_, ρ = ρ_0_) and ν_0_ = ν(*T* = *T*_0_, ρ = ρ_0_) of the corresponding equilibrium system for which *T* = *T*_0_ and ρ = ρ_0_ are uniform.

Associated with the squared deviations of the particle velocities in the cell from the velocity of the center of mass is the local temperature of a cell *T*_cell_, obtained by using kinetic gas theory and resulting in the expression
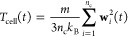
6To perform thermophoresis simulations, we employ a temperature gradient along the *z*-axis in the background solvent by thermostatting a cold slab at *z* = 0 to temperature *T*_c_ and a hot slab at *z* = *l* to temperature *T*_h_ > *T*_c_, each of thickness *a* (see Figure 1a).^32^ This establishes a linear temperature gradient **∇***T* between the two slabs with magnitude
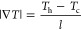
7Subsequently, in order to employ periodic boundary conditions in all 3 dimensions, we double the box size along the temperature gradient which mirrors the temperature profile in the second half of the box. As a result, the linear temperature profile is alternating in direction. Therefore, we define  as the unit vector that points in the direction of the temperature gradient **∇***T*
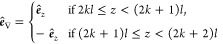
8, obtaining a periodicity of 2*l* for the periodic boundary conditions. Then we can write
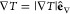
9The linear temperature profile can be described using
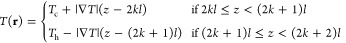
10, and has an average temperature of *T*_0_ = (*T*_h_ + *T*_c_)/2. Since the solvent is an ideal gas, the local temperature *T*(**r**) must be accompanied by a local density ρ(**r**), so that the product *k*_B_*T*(**r**)ρ(**r**) = *P*, corresponding to the fluid pressure *P* that must be constant throughout the box.^25^ This condition gives rise to a local particle density
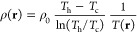
11with the average particle density ρ_0_ = ⟨*n*_c_⟩/*a*^3^. This further results in a nonuniform solvent viscosity in space (see Figure 1b). The local dynamic viscosity η(**r**) of the MPCD solvent can be calculated as the sum of the collisional contribution to the dynamic viscosity η_coll_ and the kinetic contribution η_kin_ according to^33^
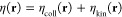
12
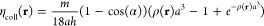
13
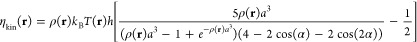
14Using the local dynamic viscosity, the local kinematic viscosity can be determined as
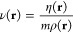
15

In order to achieve a linear temperature gradient in the MPCD fluid, we do thermostatting of the slabs using cell-level Maxwell–Boltzmann scaling.^34^ Accordingly, we draw a target kinetic energy *E*_kin_ from the probability density function
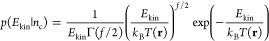
16using the target local temperature *T*(**r**), the Gamma function Γ and degrees of freedom *f* = 3(*n*_c_–1), for each cell that is part of a thermostatting slab and each time step. Note, we have to allocate the cells for thermostatting separately from, albeit methodologically identically to, the collision cells by omitting the random displacement employed for the latter to ensure Galilean invariance. Subsequently, the relative velocities are scaled by a constant κ that results from the ratio between target and actual kinetic energy in the cell
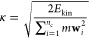
17
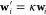
18This generates a local velocity distribution that follows a Maxwell–Boltzmann distribution with local temperature *T*(**r**). After thermostatting only slabs, a linear temperature profile between them emerges. Associated with it is a density profile, as discussed above. In Figure 1, we show both the theoretical expectations for *T*(**r**) and ρ(**r**), eqs 10 and 11, and the simulation results, displaying excellent agreement between the two.

Our reference units for length, mass, and energy are *a*, *m*, and *k*_B_*T*_0_, and they are set to *a* = 1, *m* = 1, and *k*_B_*T*_0_ = 1. From this follows the unit of time . For our simulations, we set the solvent parameters to *h* = 0.1τ, α = 2π/3, and average particle density ρ_0_ = 10/*a*^3^. If not stated otherwise, we work with a value of the temperature gradient
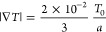
19and set the boundary temperatures *T*_h_ and *T*_c_ accordingly, depending on box size *l* and respecting that the average temperature is equal to the reference temperature *T*_0_. To save solvent equilibration time, initial positions are distributed according to the local density profile, eq 11, and initial velocities are drawn from the Maxwell–Boltzmann distribution of local temperature *T*(**r**) following the temperature profile given by eq 10. Thermostatting is only applied to the solvent. The polymer introduced below is thermostated solely indirectly through interaction with the solvent.

### 2.2 Interaction Model

The combination of thermophoretic behavior and varying solvent quality conditions that we aim at investigating requires that we introduce a great deal of flexibility in the interparticle interactions to be employed. In implicit-solvent polymer models usually employed whenever one focuses, e.g., entirely on equilibrium properties or when hydrodynamics is ignored, the solvent quality is expressed effectively only, in the form of the ensuing monomer–monomer interactions. Accordingly, repulsive forces between monomers model good or even athermal solvent conditions, whereas worsening solvent quality manifests itself into attractions of varying strength between the monomers. In the standard MPCD approach to polymer solutions, all interactions between solvent particles and between monomers and the solvent are set to zero, and the momentum exchange is carried out entirely via the collision step. As we will shortly demonstrate, for the case of thermophoresis, this is a poor choice, as it leads to a null result. In other words, explicit monomer–solvent interactions are required for thermophoresis to take place. Accordingly, we are led to introducing interaction potentials *U*_μν_(*r*) between particles of types μ and ν at separation *r*, where μ, ν ∈ {*m*, *s*} and ‘*m*’ stands for “monomer” whereas “*s*” stands for “solvent”.

The family of interaction potentials *U*_μν_(*r*) has the form of a generalized Lennard–Jones pair potential with modifiable attraction strength^35^ modeled via suitable parameters λ_μν_, namely
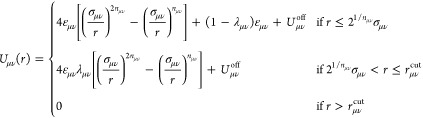
20having an offset potential  given by
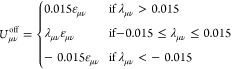
21such that the magnitude of the offset is no more than a typical offset choice for the standard Lennard–Jones potential (*n* = 6, λ = 1) at *r* = 2.5σ. Depending on the offset potential, we define the cutoff distance  as
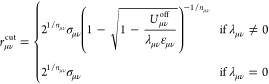
22such that the resulting potential U_μν_(*r*) is continuous, and therefore its gradient is well-defined.

In Table 1 we summarize the parameters employed for the three interaction potentials. Note that the solvent particles do not interact with one another, so that we can maintain the efficiency of the solvent-MPCD algorithm also in our case. Accordingly, ε_ss_ = 0, a choice that renders the remaining solvent–solvent interaction parameters, λ_ss_, σ_ss_, and *n*_ss_ irrelevant. Moreover, we fix the monomer–monomer interaction parameters, so that we steer both the solubility and the thermophoretic response of the polymers by changing a single quantity, λ_*ms*_, which controls the strength of the attractions between monomers and solvent molecules. For parsimony in notation, we set λ_*ms*_ ≡ λ in what follows and also *n*_*ms*_ ≡ *n*, since *n*_mm_ is fixed at the Lennard–Jones value *n*_mm_ = 6, see Table 1.

**Table 1:** Parameters for the Non-Bonded Interaction Potentials *U*_μν_(*r*) of eq 20 Acting between Monomers and Solvent Molecules

μν	ε_μν_	σ_μν_	*n*_μν_	λ_μν_
ss	0			
mm	ε = *k*_B_*T*_0_	*a*	6	0
ms	ε = *k*_B_*T*_0_	σ = 0.5*a*	*n* = 12	λ (variable)

### 2.3 Polymer Chain

We simulate fully flexible linear polymer chains consisting of *N* monomers, each with a mass *M* = 10 *m* relative to the solvent. The position vector of monomer *i* is denoted as **R**_*i*_ and its velocity as . The monomer–monomer interactions are modeled via molecular dynamics (MD) using the Kremer–Grest model.^36−38^ Specifically, we apply the purely repulsive Weeks–Chandler–Andersen (WCA) version^39^ of the potential of eq 20, acting between all monomer pairs to account for excluded volume interactions
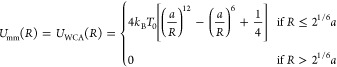
23where *R* ≡ |**R**_*i*_ – **R**_*j*_| is the magnitude of the separation vector between monomers *i* and *j*. As can be seen by comparing eqs 20 and 23, we have set the energy scale ε_mm_ ≡ ε = *k*_B_*T*_0_, the length scale σ_mm_ = *a*, the exponent *n*_mm_ = 6, and the interaction parameter λ_mm_ = 0, see also Table 1.

We further apply the finitely extensible-nonlinear-elastic (FENE) potential^38^*U*_FENE_(*R*) between consecutive monomers along the chain to simulate bonding
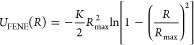
24In eq 24 above, *R* ≡ |**R**_*i*+1_ – **R**_*i*_| and 1 ≤ *i* ≤ *N* – 1. For the spring constant *K* and the maximum distance *R*_max_ between two neighboring monomers, we use the common values *K* = 30ε/σ_mm_^2^ and *R*_max_ = 1.5σ_mm_.

### 2.4 Polymer–Solvent Interaction

After establishing both the solvent environment and the polymer model, the next crucial step is to define the interaction mechanism between the polymer and solvent particles. This section will detail the methods employed to accurately represent these interactions.

Given that solvent–solvent collisions occur at discrete time intervals of *h* = 0.1τ, and our molecular dynamics operates with time intervals of Δ*t*_MD_ = 0.002τ, the MD propagation must be performed *h*/Δ*t*_MD_ = 50 times for each multiparticle collision to synchronize the system time. Additionally, we need to ensure that also the time intervals for polymer–solvent interactions are synchronized with the system time.

As we applied the mirror-inverted temperature gradient, we expanded the simulation box to double its length along the *z*-direction, i.e., the simulation box length along the *x*- and *y*-direction is *l*, whereas along the *z*-direction it is 2*l*. Depending on the polymer size, we set *l* = 30*a* for simulations that involve polymers with *N* = 20, and we set *l* = 50*a* for simulations of polymers with *N* = 50. For symmetry reasons, two polymers are placed within the simulation box, each occupying its own half-box. If either polymer approaches the end of its half-box along the *z*-direction, the simulation is terminated prematurely. This measure ensures that the polymer moves within a linear temperature gradient throughout the simulation, and consequently, it also prevents direct interaction between the two polymers.

#### 2.4.1 Interaction via Collision Coupling

One well-established and simple method to simulate polymer–solvent interaction is to include the monomers in the multiparticle collision step.^40^ This ensures exchange of momentum between polymer and solvent, while avoiding computationally expensive pair-calculations of forces. For this purpose, the calculation of the center-of-mass velocity in the cell **v**_cm_ from eq 4 is modified to
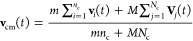
25with the monomer velocities **V**_*j*_ and the number of monomers *N*_c_ in the cell. The multiparticle collision, eq 5, is then applied to both solvent particles and monomers, but using the joint center of mass velocity **v**_cm_(*t*) from eq 25.

**Figure 2:**
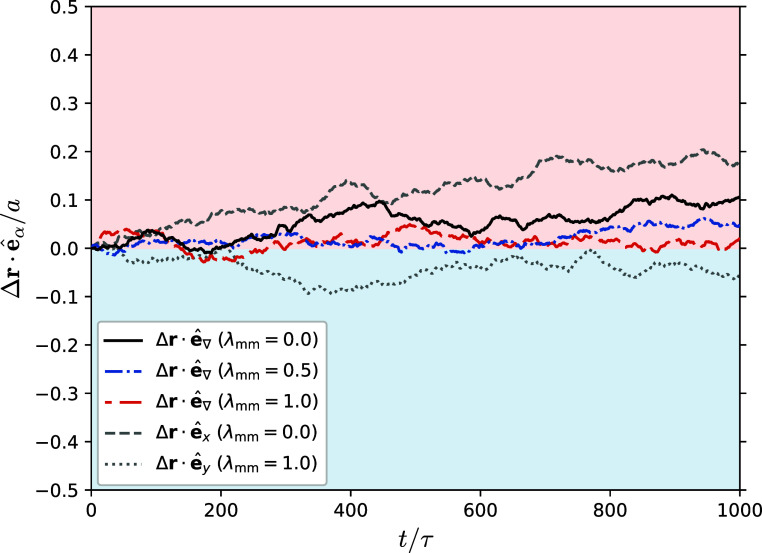
Displacement of the polymer center-of-mass along the box axes over time using collision coupling. The temperature gradient is applied along the *z*-axis, where the red background in the upper half of the graph indicates the hotter side, and the blue background in the lower half indicates the colder side (along the *x*-axis and *y*-axis the temperature is constant). All curves show an average over 480 runs with two polymers each.

Performing simulations using this conventional method of coupling through collision and applying the temperature gradient in the background solvent, brings about no thermophoretic motion of the polymer chain at all, as can be seen in the results shown in Figure 2. Indeed, we have tested the thermophoretic behavior of polymer chains with different monomer–monomer interactions, by modifying the interaction parameter λ_mm_ that represents different solvent qualities and thus different polymer conformations. As shown in Figure 2, there is no significant difference in the center-of-mass displacement along the temperature gradient axis, versus the *x*-axis or *y*-axis when using multiparticle collision coupling between monomers and solvent. For every choice of λ_mm_, the polymer responded in a thermophoretically neutral fashion. This hints to us that in order to study the thermophoretic behavior of polymers, we need to employ explicit monomer–solvent interactions, as discussed in Section 2.4.2.

#### 2.4.2 Interaction via Pair-Potentials

A different approach to polymer–solvent interactions, compared to the procedure described in Section 2.4.1, is applying pair potentials between monomers and solvent particles explicitly.^41^ In this work, we apply the generalized Lennard–Jones pair potential, eqs 20–22 introduced in Section 2.2. Consequently, instead of the ballistic movement, the solvent is propagated using molecular dynamics following the velocity verlet algorithm, eqs 34 and 35 below, with the same time step Δ*t*_MD_ = 0.002τ as used for monomers. Solvent particles that are positioned very far from any monomer, i.e., outside the range of the monomer–solvent forces **f**_*j*_ on monomer *j*, see eq 31, are not subjected to any force field. Therefore, solvent particles distant from monomers are propagated ballistically as in the original MPCD algorithm, but with a time-step Δ*t*_MD_ instead of *h*. Given the MPCD time step *h* = 0.1τ, additionally to MD, the solvent performs the collision step of the MPCD algorithm every *h*/Δ*t*_MD_ = 50 MD steps.

**Figure 3:**
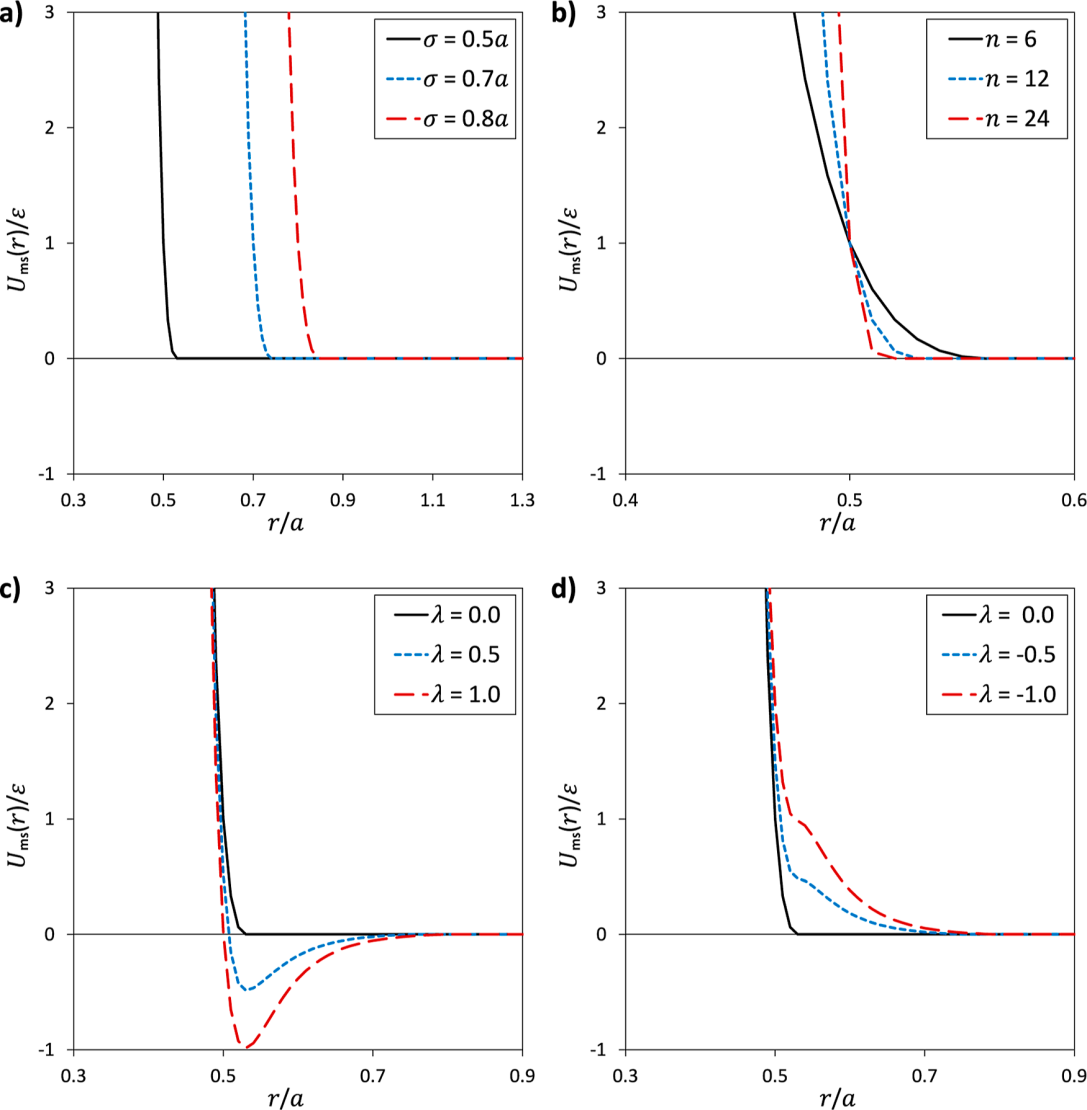
(a) Interaction potential *U*_ms_(*r*) for different effective diameters σ while the other parameters (λ = 0, *n* = 12) are fixed. (b) *U*_ms_(*r*) for different exponents *n* with fixed σ = 0.5*a* and λ = 0. (c) *U*_ms_(*r*) for different (positive) interaction parameters λ, while σ = 0.5*a* and *n* = 12 are fixed. (d) *U*_ms_(*r*) when using negative interaction parameters λ (σ = 0.5*a* and *n* = 12 fixed).

In Table 1 we summarized our choice of parameters for the monomer–solvent interaction potential *U*_ms_(*r*), which contains three parameters: the exponent *n*_*ms*_ ≡ *n*, the attraction depth λ_ms_ ≡ λ and the steric diameter σ_ms_ ≡ σ. The choices we made for *n* and λ are interconnected and we will present the procedure we followed for determining their values in Section 3; we anticipate our choices there by stating that we fixed *n* = 12 for varying values of λ and explain below our choice for σ, while in Figure 3 we show some characteristic examples of the influence typical parameter values have on the form of the interaction *U*_ms_(*r*).

Increasing the effective diameter σ increases the excluded volume, and therefore the distances between monomers and solvent particles. Accordingly, σ serves as a means to influence solvent quality, as increasing its value makes it unfavorable to find solvent particles close to the polymer and thus it can lead to collapsed polymer conformations. For fixed values *n* = 12 (or *n* = 6) and λ = 0, an effective diameter of σ = 0.5*a* corresponds to expanded polymer conformations, characterizing the good solvent regime. Increasing the effective diameter to σ = 0.8*a* (effectively giving the solvent particles an enhanced excluded volume in relation to monomers) corresponds to compact polymer conformations, characterizing poor solvent quality. While the effective diameter σ can thus be used to steer solvent quality, it did not influence the thermophoretic velocity of the polymer, which for the chosen parameters is directed toward the hot regime (see Supporting Information). Therefore, we fixed σ to the value σ = 0.5*a* and opted for steering of both the polymer conformations and the thermophoretic behavior through the single parameter λ that turned out to be a very efficient tool in steering both types of behavior independently, as will be demonstrated in Section 4.

### 2.5 Integration of the MD-MPCD Equations of Motion and Collision Rules

Consider a system containing *N* monomers (i.e., a single chain in our case) as well as *n*_s_ solvent molecules. Define as {**R**^*N*^}, {**V**^*N*^} the collective coordinates and velocities of the monomers and as {**r**^*n*^_s_}, {**v**^*n*^_s_} the corresponding quantities for the solvent molecules, respectively. According to the preceding discussion, the interactions between all particles involved are governed by potential energy functions Φ_μν_({**R**^*N*^},{**r**^*n*^_s_}) as follows
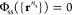
26
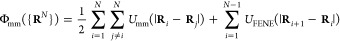
27and
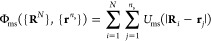
28The total potential energy function Φ({**R**^*N*^},{**r**^*n*^_s_}) is given as the sum
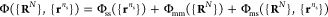
29allowing us to obtain the forces **F**_*i*_ on monomer *i* and **f**_*j*_ on solvent particle *j* as
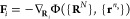
30and
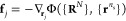
31Integration of the MD trajectories is now performed using the velocity Verlet scheme,^42−44^ which reads as
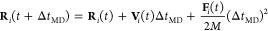
32
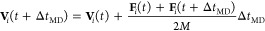
33for the position and velocity of monomer *i*, 1 ≤ *i* ≤ *N*, and
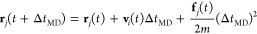
34
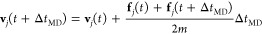
35for solvent particle *j*, 1 ≤ *j* ≤ *n*_s_. Finally, after completing 50 MD steps, the solvent particles solely are subjected to the MPCD collision rule described by eqs 3–5.

## 3 Simulations at Constant Temperature: Tuning Solvent Quality

The introduction of explicit monomer–solvent interactions has ramifications not only for the thermophoretic behavior but also for the equilibrium polymer conformations and sizes. As the latter are crucial for the polymer behavior, and they also possibly correlate with thermophoresis, we first analyze the equilibrium properties of the model proposed in Section 2.4 in order to characterize the consequences of the specific polymer–solvent interactions. We focus on the classification of solvent quality using the radius of gyration *R*_G_ of the polymer. The instantaneous value for every conformation, , is given by the expression^45^
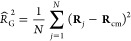
36whereas the radius of gyration *R*_G_ follows as a root-mean-square value after an average ⟨···⟩ over all conformations, as
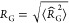
37In eq 36 above, **R**_cm_ = 1/*N*∑_*i*=1_^*N*^**R**_*i*_ denotes the position of the polymer’s center of mass.

**Figure 4:**
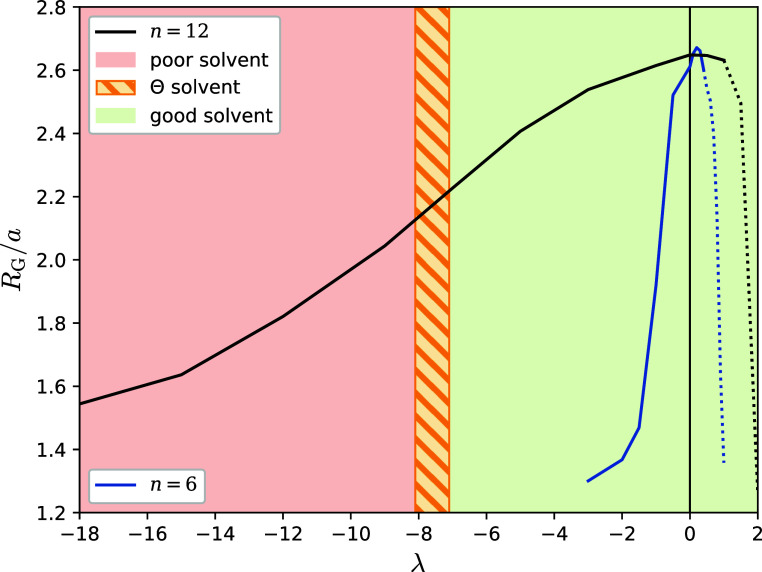
Radius of gyration  scaled over the monomer diameter *a* against interaction parameter λ for a linear chain polymer with *N* = 20. The black curve shows the polymer with an interaction potential exponent *n* = 12, while the blue curve depicts *n* = 6. The good solvent regime of the polymer with *n* = 12 is highlighted by a green background, the poor solvent regime is highlighted in red. The golden bar in the middle shows the area in which the Θ point of the polymer for *n* = 12 is located. The dotted curves represent nonphysical regimes for the interaction parameter λ due to solvent accumulation, as explained in the text.

The polymer conformation is mainly controlled by the monomer–solvent attraction strength λ in conjunction with the exponent *n* of the corresponding interaction *U*_ms_(*r*). The exponent *n* there controls the steepness of the potential, representing soft particles for small *n* and hard spheres for *n* → ∞ (see, e.g., Figure 3b). Consequently, the range of interaction increases due to the softening of the potential by decreasing *n*. By changing *n* and therefore the range of interaction, we can tune the sensitivity of the variation of the radius of gyration *R*_G_ of the polymer toward modification of the interaction parameter λ, making the polymer very sensitive to changes in λ when using small *n* = 6. In Figure 4, we show the influence of the interaction parameter λ on the radius of gyration *R*_G_ when using *n* = 12 compared to that when employing *n* = 6. It can be seen that for *n* = 12, the gyration radius changes much more slowly with λ compared to *n* = 6. Such a slow variation is a desirable feature, as it allows us to explore wide conformational changes ranging from good to poor solvent in a convenient way. Otherwise, however, a modification of *n* has no drastic physical consequences, and thus the choice *n* = 12 we made does not restrict the generality of our approach.

Modifying the interaction parameter λ can be used to tune the radius of gyration *R*_G_ of the polymer (see Figure 4) directly. For λ = 0, the monomer–solvent potential is purely repulsive. It corresponds to the standard truncated and shifted Lennard–Jones potential but with tunable exponent *n*. Even though monomer–solvent interactions are purely repulsive, the polymer adopts an expanded state. This is because the monomer–monomer interactions we are using are purely repulsive as well. By setting λ < 0 (Figure 3d), we enter the regime of enhanced solvent–monomer repulsions. This causes the polymer to adopt compact conformations, eventually leading to the poor solvent regime for sufficiently low λ, due to the high repulsion between solvent particles and monomers, exceeding the effects of the direct monomer–monomer repulsions.

**Figure 5:**
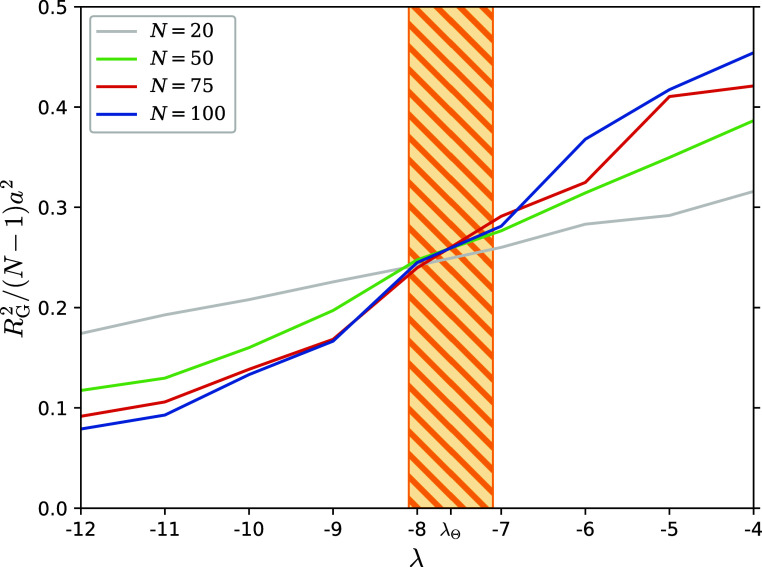
Θ Point is located at the λ = λ_Θ_ ≈ −7.6 for which all curves of *R*_G_^2^/(*N* – 1) for different and sufficiently high polymerization degrees *N* fall on top of each other. Note, for *N* = 20 (gray) the polymer is too short to expect good scaling behavior.

**Figure 6:**
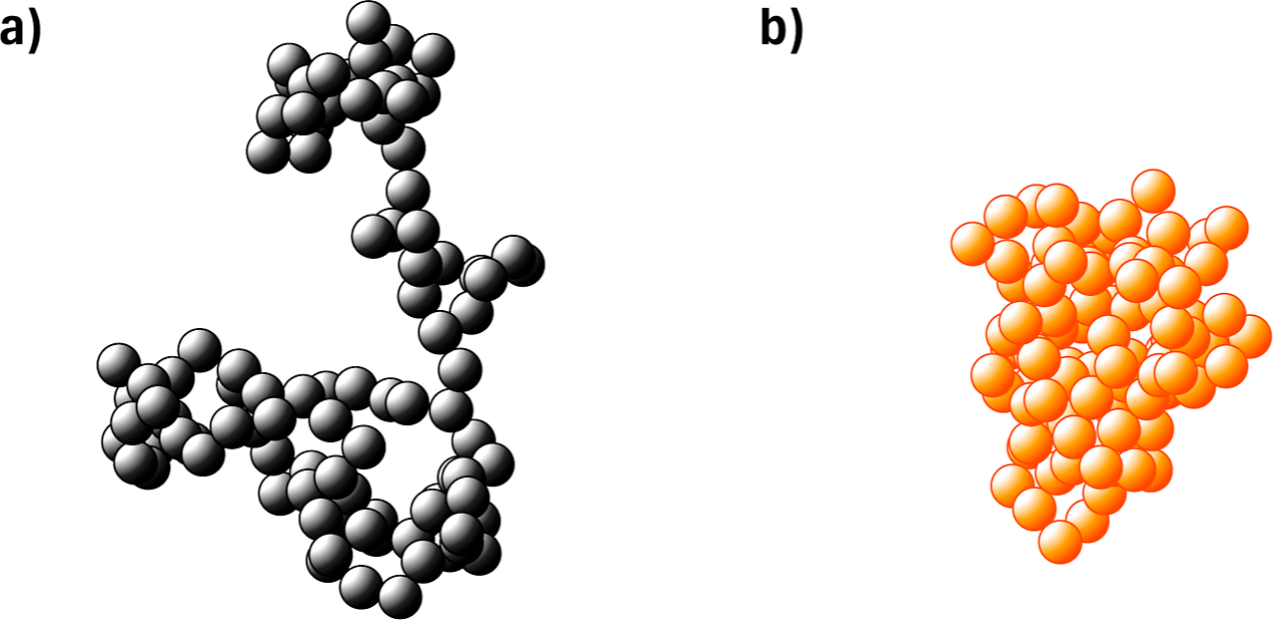
Example snapshots of a polymer with *N* = 100 in (a) a good solvent state, here with λ = −4, and (b) a poor solvent state, here with λ = −14.

In-between the good and poor solvent conditions lies the Θ-point, at which the polymer shows ideal scaling of its size,^45^ i.e., *R*_G_^2^ ∝ (*N* – 1), with the number of bonds *N* – 1 of a polymer with degree of polymerization *N*. To determine the value λ_Θ_ of the parameter λ that leads to Θ-like behavior, we analyzed how the radius of gyration *R*_G_ depends on the interaction parameter λ for polymers of different polymerization degrees *N*; in particular, we plot in Figure 5 the quantity *R*_G_^2^/(*N* – 1) against λ for various *N*-values, to identify the Θ point as the point where all curves of different *N* meet. Apart from the lowest *N*-value (which is indeed rather small, *N* = 20), the other curves all cross at λ_Θ_ ≅ −7.6, which is thereby identified with the Θ-point of the model; we also arbitrarily denote the whole region −8.1 ≤ λ ≤ −7.1 Θ-like and we denote it with the cross-hatched band in Figure 5. For λ < λ_Θ_ the polymer enters the poor solvent regime, while for λ > λ_Θ_ the polymer is in a good solvent state. Characteristic conformations for both regimes are shown in Figure 6.

**Figure 7:**
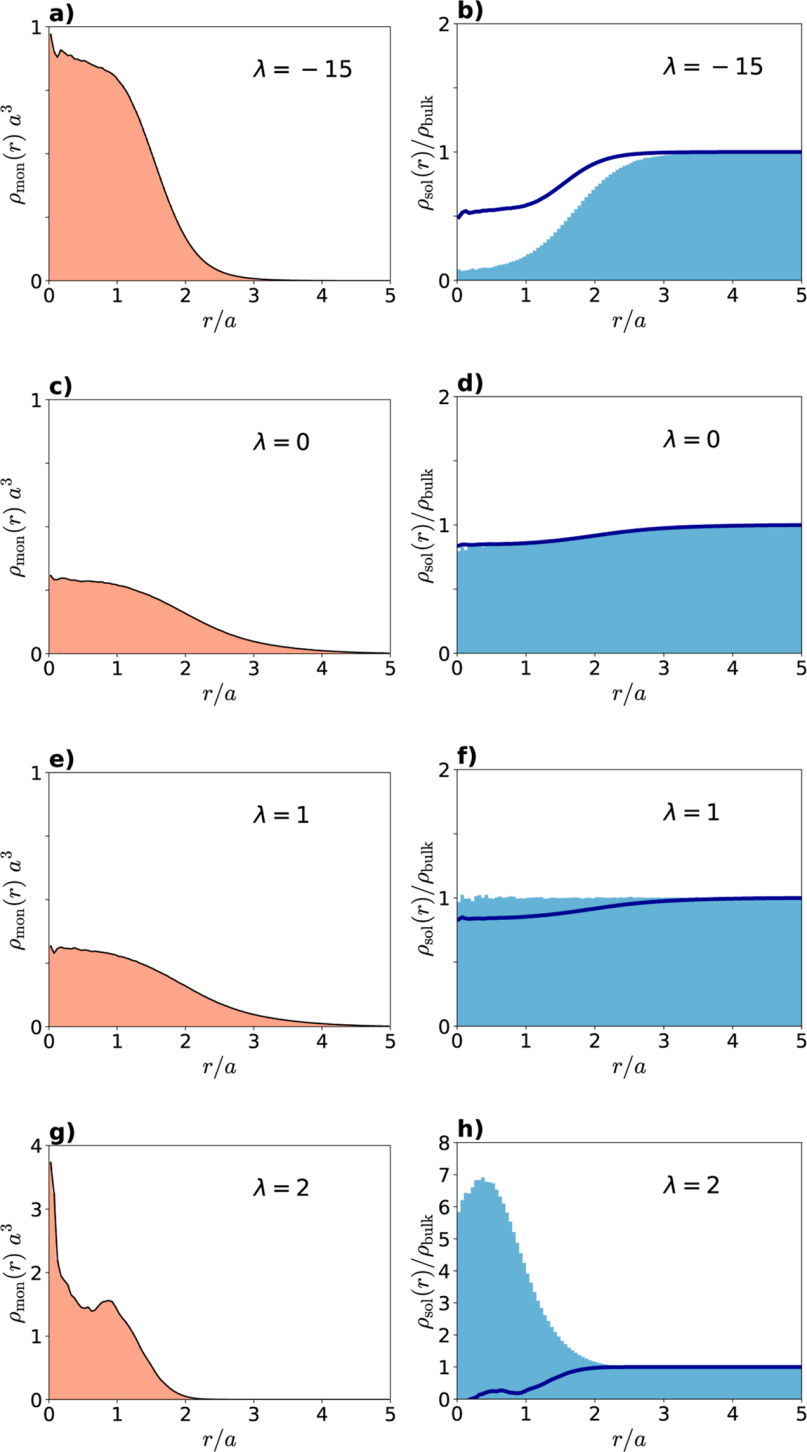
Radial monomer density ρ_mon_(*r*) histograms (orange) and the corresponding radial solvent density ρ_sol_(*r*) histograms (blue) with respect to the polymer center-of-mass for different interaction parameters λ, as indicated on the titles of panels (a–h). The thick blue curves are obtained from eq 39. Simulation results are averages over 480 runs, i.e., for 960 polymers.

Particular attention needs to be paid in the region λ > 0, which implies attractions between monomers and the solvent. Whereas one would naively expect this condition to correspond to even better solvent conditions, the results marked with dotted lines in Figure 4 show that the attractive potential between monomer and solvent does not lead to polymer expansion, as one may expect, but again forces the polymer into a collapsed state for sufficiently large values λ. This is a consequence (artifact) of the ideal nature of the solvent; to understand it better, we take a closer look at the radial density function of monomers ρ_mon_(*r*) relative to the polymer center-of-mass. By definition, this quantity fulfills the condition
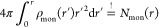
38where *N*_mon_(*r*) is the cumulative monomer number from the center-of-mass at *r*′ = 0 up to *r*′ = *r*. Accordingly, *N*_mon_(*r* → ∞) = *N* is the total monomer number. Assuming further that solvent and monomers pack in an incompressible fashion (which is manifestly not true in the absence of solvent–solvent steric interactions), and setting ρ_bulk_ to be the value of the solvent density far away from the region occupied by the monomers, we can define the theoretical radial solvent density function ρ_sol_(*r*), normalized by the bulk solvent density, through the monomer density function via
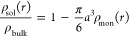
39Our goal is to cross-check to which extent the simulation results for ρ_sol_(*r*) conform to eq 39 above and what is the origin of possible large deviations from it. The radial density functions of monomers and solvent particles for various interaction parameters λ are shown in Figure 7. Based on the measured monomer profiles, depicted in Figure 7a,c,e, and g, the solvent densities ρ_sol_(*r*) expressed by eq 39 are depicted as thick blue solid lines in the corresponding panels in Figure 7b,d,f,g, for the value of λ shown on each panel.

For poor solvent conditions, λ = −15 shown in Figure 7a,b, the situation is physically reasonable: the monomer density profile shows a compact polymer conformation with an enhanced core, whereas the solvent is depleted from the polymer region, as is indeed the case for poor solvents. The discrepancy between the measured solvent profile, light blue region in Figure 7b, and eq 39, thick blue line in the same, is physically inconsequential: the solvent is in reality even more depleted from the polymer’s interior than what eq 39 predicts but this causes no problems and it can be traced back to the broad range of the solvent–monomer repulsion for such a strongly negative λ-value.

For the good-solvent case, λ = 0, shown in Figure 7c,d, the solvent takes up exactly the space left from monomer excluded volume. Once again, things make sense since now the polymer is more extended, the monomer density is correspondingly broader and lower than in the case λ = −15, and the solvent mixes well with the monomers in the polymer’s interior. Furthermore, as shown in Figure 7e,f, a slightly attractive potential (λ = 1) draws more solvent particles into the polymer center. Resulting into a solvent distribution that is nearly uniform, as is the case for the usual MPCD solvent particles, which are ideal not only with respect to one another but also with respect to the monomers.

Things get drastically different for even more positive λ-values, as shown in Figure 7g,h pertaining to λ = 2. Here, the polymer adopts a collapsed state where a pronounced shell of first neighbors is apparent, indicating even crystalline order. Moreover, the solvent strongly accumulates near the polymer center, exceeding by far the theoretical curve. It is straight forward to conclude that the polymer collapse in this regime is due to the missing excluded solvent–solvent-volume. For strong solvent–polymer attractions (λ > 1 implies an attraction minimum deeper than the thermal energy *k*_B_*T*), the attraction of solvent particles toward monomers exceeds the solely entropic incentive for solvent particles to distribute evenly, and hence the solvent particles accumulate near the polymer to benefit from the low-energy local environment. In the absence of steric hindrance between the solvent particles, this accumulation is becoming further enhanced. As a result, the polymer collapses to wrap around the center of accumulation and also benefit from the attractive interaction. This is an unphysical behavior, stemming from the point-like nature of the MPCD solvent; in real fluids, solvent particles have excluded volume interactions preventing accumulation. Since the polymer collapse is here only a consequence of the unphysical solvent accumulation, but we wish at the same time to maintain the computational efficiency offered by the ideal character of the MPCD solvent, we restrict our range to λ ≤ λ_max_ = 1, where the solvent density is still physically meaningful.

**Figure 8:**
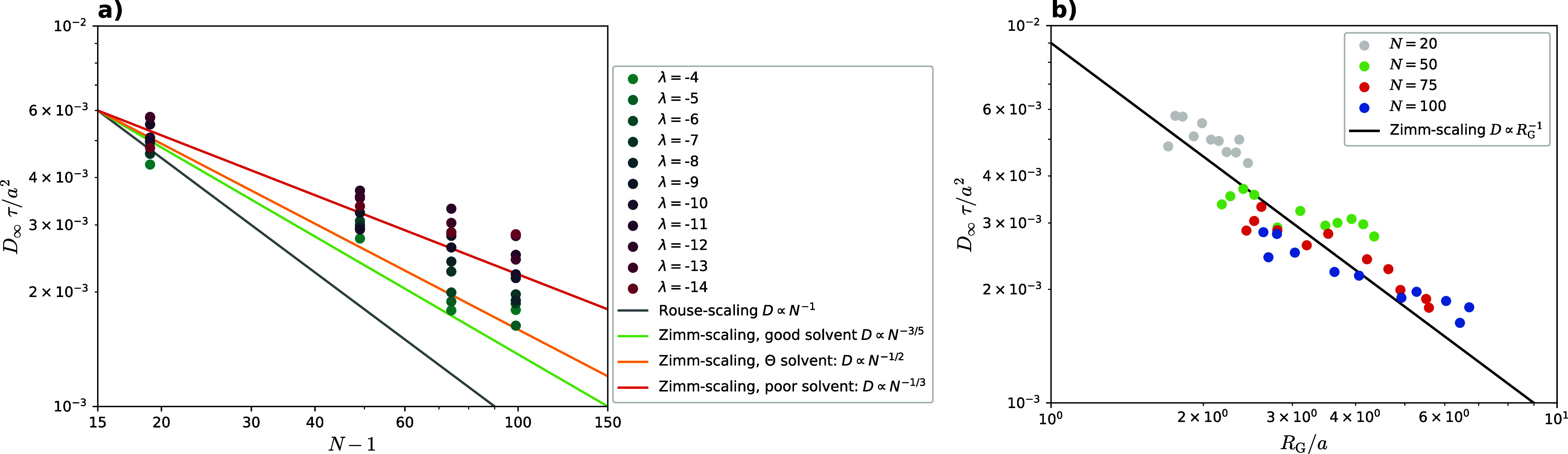
(a) Diffusion coefficient *D*_∞_ against the number of bonds *N* – 1. As given by eq 42, polymers of different *N* that share the same Flory exponent ν should follow the same scaling. As a reference, 3 lines that are representative of Zimm-scaling are given in red (poor solvent, ν = 1/3), yellow (Θ solvent, ν = 1/2), and green (good solvent, ν = 3/5). All polymer scaling curves should fall somewhere in this range. In contrast, Rouse-scaling is shown by the gray line and should not be followed by any polymer data. (b) Relationship between diffusion coefficient *D*_∞_ and radius of gyration *R*_G_. According to eq 43, all points should follow the same scaling represented by the black line.

In order to understand how the polymer is affected by hydrodynamic interactions in the proposed model, we investigate the scaling of the diffusion coefficient *D* in dependence of the degree of polymerization *N*. To calculate *D*, we look at the mean squared displacement of the center-of-mass  over time. The diffusion coefficient is then extracted from the slope according to^45^
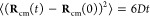
40The diffusion coefficient is subject to finite size effects, that originate from the limited box size *l*. Because hydrodynamic interactions are of long-range, we need to account for this and apply a finite size correction. To calculate the infinite size diffusion coefficient *D*_∞_, a well established method is to apply the first order correction following^46,47^
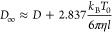
41using the solvent viscosity η as given by eqs 12–14. If the model produces dynamics that are in agreement with Zimm theory, i.e. it correctly reproduces hydrodynamic interactions, then the diffusion coefficient is supposed to scale with the number of bonds *N*–1 following^45^
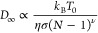
42with the Flory exponent ν. For a Θ solvent, the Flory exponent is ν = 1/2. For poor solvent and good solvent quality, the Flory exponent can be estimated to ν = 1/3 and ν = 3/5 respectively. In contrast, if the model produces dynamics following Rouse theory, i.e. it ignores hydrodynamic interactions, we would expect *D*_∞_ ∝ 1/*N*. Figure 8a shows, that the diffusion coefficient clearly follows Zimm-scaling, as it should. We can rewrite eq 42 to examine the scaling of the diffusion coefficient with the radius of gyration, using the relationship *R*_G_ ∝ (*N* – 1)^ν^ to obtain
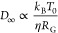
43This enables us to join all data points, independent of solvent quality, into one plot. Figure 8b shows that data points for different *N* and different λ all fall on the same curve, representing Zimm-scaling.

## 4 Thermophoresis of Polymers

**Figure 9:**
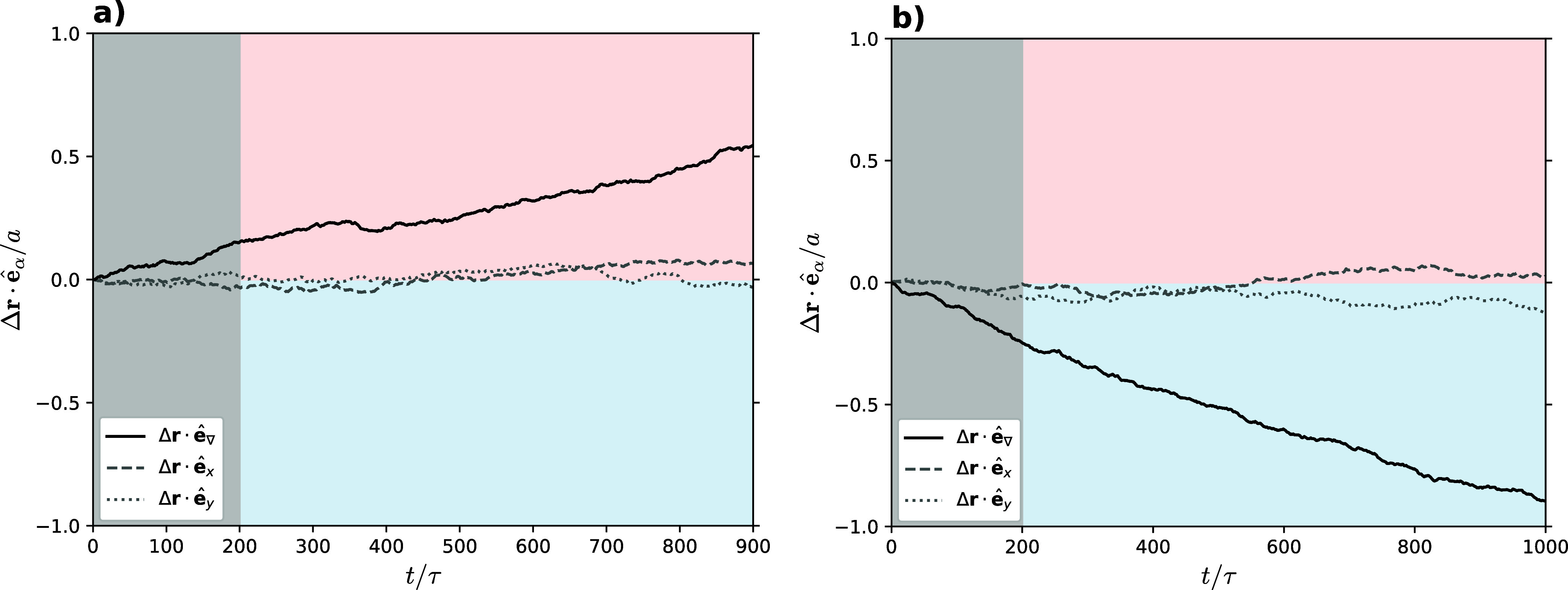
Ensemble-averaged displacement of the polymer center-of-mass along the box axes over time by modeling monomer–solvent interaction via pair-potentials. (a) A polymer that has monomer–solvent interactions with enhanced repulsion, λ = −1. The displacement along the temperature gradient axis toward the hot side is well pronounced. (b) A polymer with attractive monomer–solvent interactions, λ = 1. Also here, the displacement along the temperature gradient has a clearly pronounced preferred direction, in this case toward the cold side. Both show an average over 480 runs with 2 polymers of *N* = 20.

Having completed the analysis of the conformational and equilibrium dynamic properties, we proceed here to thermophoresis. Our goal is not only to examine the phoretic behavior but also to explore how it correlates with solvent quality. According to eq 1, the thermophoretic mobility *D*_T_ can be calculated from the thermophoretic drift velocity **v**_T_. Using our notation with the unit vector along the temperature gradient gradient , we rewrite eq 1 as
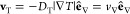
44introducing the velocity along the temperature gradient *v*_∇_ = −*D*_T_|**∇***T*|. Using this notation, the case *v*_∇_ > 0 represents a thermophilic polymer, while *v*_∇_ < 0 means that the polymer is thermophobic, independent of the temperature gradient direction with respect to the box. *v*_∇_ is obtained by calculating the slope of the polymer center-of-mass displacement Δ**r** along the temperature gradient over time from an ensemble as
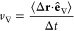
45Because of thermal fluctuations and relatively small drift velocities, we need to average over 480 simulation runs. This becomes evident when we look at the Peclet number of our system. The Peclet number of a polymer can be calculated following^17,48^
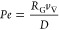
46and gives the ratio between directed and diffusive motion. It takes a polymer specific length scale, for which we use the radius of gyration *R*_G_. A polymer simulated in our system with *N* = 20 has a diffusion coefficient in the order of *D* ∼ 5·10^–3^*a*^2^/τ (see Figure 8a), a radius of gyration of *R*_G_ ∼ 2*a* (see Figure 4), and a thermophoretic drift velocity in the order *v*_∇_ ∼ 10^–3^*a*/τ (as later shown by Figure 11). This results in a Peclet number of *Pe* ∼ 0.4. It thus becomes evident that our simulation is dealing with the low-Peclet-number regime, in which the concept of local thermodynamic equilibrium can be applied and the proportionality relation between thermophoretic velocity and temperature gradient holds.^17^ Accordingly, the molecule, while being driven through the temperature gradient, is also subject to incessant and strong thermal fluctuations, present in the MPCD-scheme through the random collision step of the algorithm. Indeed, the drift curves shown in Figure 9 display visible noise-like features, despite the fact that they have been averaged over 480 runs with 2 polymers; individual trajectories (not shown) have strong noise-induced fluctuations. We also emphasize that there are no body-forces acting on our polymers, which are neutral, and thus they are not dragged by additional, external electric fields, in contrast to charged colloids or DNA-segments often employed experimentally.^17−19^

We use equilibrated polymer conformations and initialize the solvent according to its density profile along the gradient, nevertheless, we still ignore the first Δ*t* = 200τ segment of the simulation to give the system additional equilibration time for local solvent density adjustments before the thermophoretic drift stabilizes.

### 4.1 Thermophoresis of Homopolymers

In Figure 9 we show the ensemble-averaged center-of-mass displacement of homopolymers in a temperature gradient using interaction potentials between monomer–solvent pairs with λ = −1, Figure 9a, and λ = 1, Figure 9b.

**Figure 10:**
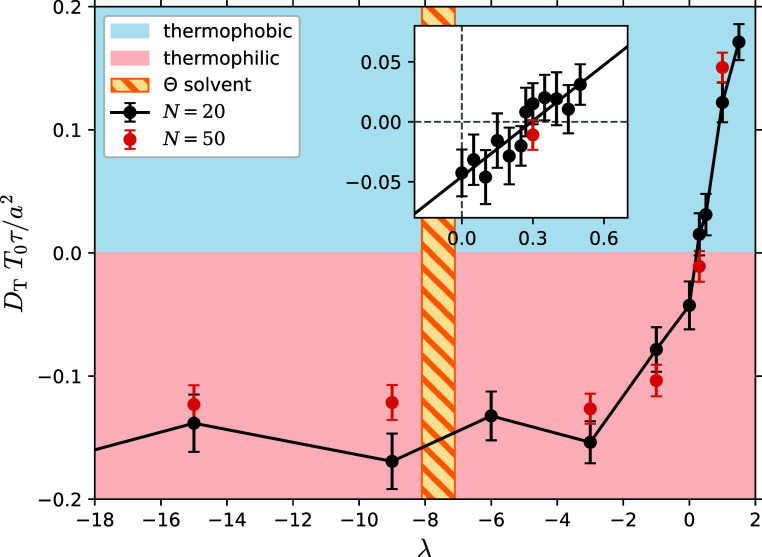
Thermophoretic mobility *D*_T_ is shown for different interaction parameters λ. λ ≤ 0 represent purely repulsive monomer–solvent interactions and lead to thermophilic polymer behavior (red region). Error bars are estimated using the standard deviation of the mean for the ensemble average. As we add attraction to the monomer–solvent interaction (λ > 0), the polymer shifts from being thermophilic to thermophobic (blue region). The Θ point regarding solvent quality is found at λ_Θ_ ≈ −7.6, represented by the dashed yellow bar. Polymers with size *N* = 20 are visualized by black points, size *N* = 50 are shown in red. The inset shows the region around λ = 0 in more detail. Here we see that the point at which polymers behave thermophoretically neutral is located at λ_*_ ≅ 0.3.

A comparison of the findings in Figure 9 with the displacement curves in Figure 2, obtained by using interaction via collision coupling, reveals that a pronounced thermophoretic drift only emerges when employing explicit solvent–monomer interaction potentials. To analyze how the thermophoretic effect depends on polymer–solvent interactions, we perform simulations of homopolymers with different interaction parameters λ and compare their thermophoretic mobilities *D*_T_ in Figure 10. We find that a purely repulsive monomer–solvent interaction potential (λ ≤ 0) always results in thermophilic polymer behavior, i.e., polymer movement toward the hot side. We attribute this to the fact that a polymer that repels solvent will avoid regions with high solvent density, and move to regions with lower solvent density, which corresponds to the hot side of the simulation box. Interestingly, the thermophoretic mobility approaches a plateau value for λ ≲ −3. We attribute this effect to the depletion of solvent from the polymer core, therefore minimizing interaction between the two. Adding attraction to the monomer–solvent potential (λ > 0) makes the polymer less thermophilic toward the solvent, even changing its behavior to being thermophobic, i.e., polymer movement to the cold side, given that the attraction is sufficiently strong. This we rationalize by the fact that a polymer that attracts solvent favors moving to regions with higher solvent density, i.e., to the cold side. We emphasize that the tipping point in which the polymer is neutral toward the temperature gradient does not occur at λ = 0, but rather at λ_*_ ≈ 0.3, where the potential is already weakly attractive.

Experimental research by de Gans et al.^11^ and Kita et al.^12^ on solutions of poly(ethylene oxide) (PEO) showed very similar behavior. Both works measured the thermophoretic mobility of PEO in a mixture of water and ethanol with varying composition. For PEO, water is a good solvent with strongly attractive interactions over hydrogen bonding, while in pure ethanol, PEO is insoluble. As a result, they found that PEO behaves thermophobic if the water mass fraction in the solvent is high. As they increase the ethanol fraction in the solvent, they observe a sign change in *D*_T_ and PEO starts to behave thermophilic. The tipping point in which PEO is neutral toward the temperature gradient is observed for a solvent composition of w_*_ = 83% water in ethanol.^11^ de Gans et al. further measured the Θ point of PEO as a function of water mass fraction in the solvent, which turned out to be at *w*_Θ_ ≈ 18%, far away from the tipping point where the sign change occurs and well within the thermophilic regime. This agrees very well with our findings, where the tipping point lies well within the good solvent regime, and *D*_T_ > 0 is found only for strongly attractive polymer–solvent interactions. Additionally, the curve by Kita et al. in which they plot *D*_T_ against water mass fraction looks very similar to our Figure 10, including even the plateau region in the thermophilic regime.^12^ Experimental research involving the thermophoretic behavior of poly(*N*-isopropylacrylamide) in ethanol (a good solvent) with varying average temperature also found the sign change of *D*_T_ to occur well within the good solvent regime,^13^ therefore being in agreement with our results. However, experiments with polystyrene (PS) in a cyclohexane/toluene solvent mixture did not show a sign change in *D*_T_ by varying the solvent composition, even though toluene is classified as a good solvent for PS, and cyclohexane is a Θ solvent.^49^ Therefore, we conclude that the tipping point in which the sign change of *D*_T_ occurs, is not necessarily located within the good solvent regime.

In Figure 10, we compare results for linear chains with different *N*, namely *N* = 20 and *N* = 50. It can be seen that the same monomer–solvent interaction parameter leads to the same thermophoretic mobility *D*_T_ of the polymer, independently of *N*, as observed previously in experiments.^2^ This suggests that thermophoresis is a monomer property and not a property of the polymer conformation. The fact that the Θ point of our polymer is at λ_Θ_ ≅ −7.6, which lies inside the plateau region for the thermophoretic mobility *D*_T_, additionally shows how polymer conformation and thermophoretic behavior are not correlated in a simple way, and therefore further strengthens the argument that thermophoresis is a monomer property. Simulations with σ as the free parameter also exhibit this decoupling between solvent quality and thermophoretic behavior (see Supporting Information).

**Figure 11:**
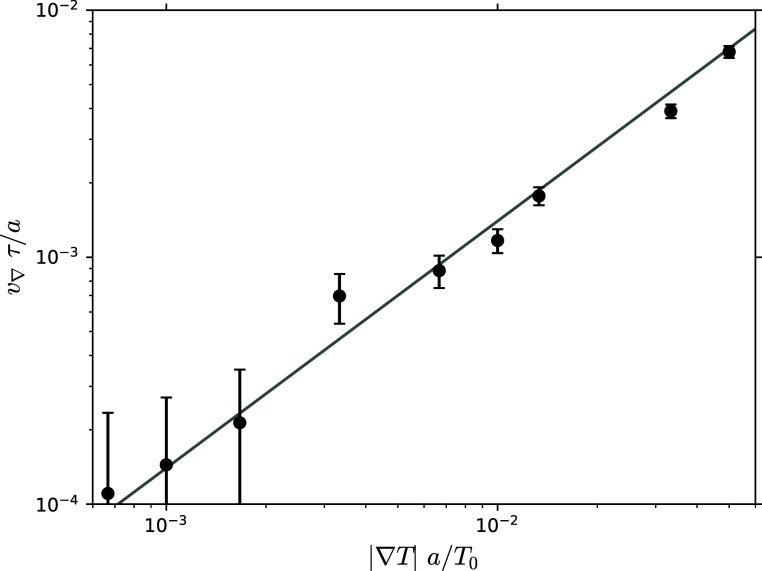
Linear relationship between thermophoretic velocity along the temperature gradient *v*_∇_ and temperature gradient |**∇***T*| is consistent over 2 orders of magnitude. The black dots show simulation results, while the gray line in the background gives a reference for linearity. Results were obtained by averaging over 480 runs, i.e. 960 polymers, using a polymer with length *N* = 20 and interaction parameter λ = −6. Error bars are estimated using the standard deviation of the mean for the ensemble average. For this parameter choice, the polymer is thermophilic, hence *v*_∇_ > 0.

We have furthermore investigated the linear regime using a polymer with *N* = 20 and λ = −6 under the influence of different strengths of temperature gradients |**∇***T*|. According to eq 1, the observed thermophoretic velocity is supposed to linearly grow with |**∇***T*|. Indeed, the results in Figure 11 confirm that the linear regime holds for the entire 2 orders of magnitude we employed, and the temperature gradient that we used for all simulations, |**∇***T*| = 2/3 × 10^–2^*T*_0_/*a* lies well within that regime. Note, for very low temperature gradients, the fluctuations dominate.

### 4.2 Thermophoresis of Block Copolymers

**Figure 12:**
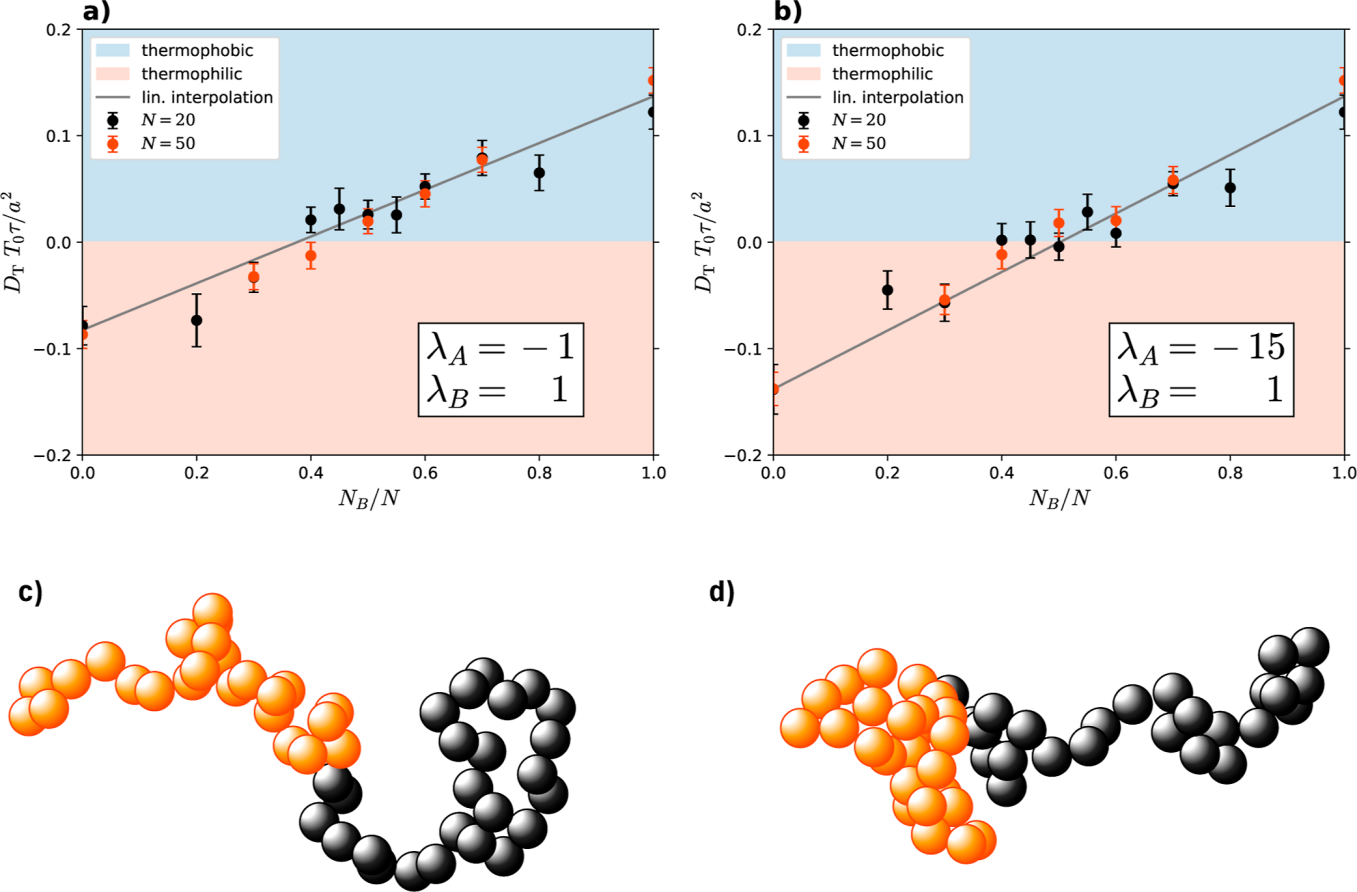
(a) Thermophoretic mobility *D*_T_ of a block copolymer that is made from 2 blocks: one block of monomer type A with λ_A_ = −1.0, the other block of monomer type B with λ_B_ = 1.0. (b) Thermophoretic mobility *D*_T_ of a block-copolymer that is made from one block with λ_A_ = −15.0, and the other block with λ_B_ = 1.0. For both, black dots show the results of a polymer with *N* = 20, red dots represent the longer polymer with *N* = 50. Error bars are estimated using the standard deviation of the mean for the ensemble average. (c,d) Snapshots for a block copolymer of *N*_B_/*N* = 0.5 with the corresponding interaction parameters used in (a,b) respectively. Orange spheres represent type A monomers, black spheres represent type B monomers.

After investigating the thermophoretic effect on homopolymers, we built block copolymers with one block made of monomers type A that are characterized by interaction parameter λ_A_ and the other block consisting of monomers type B with interaction parameter λ_B_. All other parameters were kept the same for both blocks. Varying the block-size ratio, we found that the thermophoretic mobility of block copolymers behaves linearly on the relative block size *N*_B_/*N*, and interpolates between the two extremes representing the corresponding homopolymers with 100% A and 100% B monomers, see Figure 12. This further reiterates the hypothesis that thermophoresis is a monomer property. Nonsurprisingly, the thermophoretic mobility is the same for both shorter (*N* = 20) and longer (*N* = 50) block copolymers of the same type.

Keeping the interaction parameter of the one block with monomer type B at λ_B_ = 1.0, we find interpolating behavior for both choices λ_A_ = −1.0, Figure 12a and λ_A_ = −15.0, Figure 12b of the other block. The reason why this is not obvious is that the two different choices for λ_A_ lead to completely different conformations of the copolymer. While for λ_A_ = −1.0 both blocks assume an expanded state, for λ_A_ = −15.0 only block B is expanded and block A assumes a collapsed form. This could have made block B shield the solvent from block A (which has repulsive interaction toward the solvent) and therefore play the dominating role in determining the thermophoretic mobility overall. However, it turns out that the conformation did not play a significant (enough) role here, and the total thermophoretic mobility is only determined by the ratio between the number of monomers type A to monomers type B.

## 5 Conclusions and Outlook

In this work we found that in MPCD computer simulations, thermophoresis of polymers is achieved only when monomer–solvent potentials are accounted for explicitly. Therefore, we have introduced a new type of pair potential in the form of a generalized Lennard–Jones potential that is characterized by the interaction parameter λ and governs both the solvent quality and the thermophoretic mobility of the polymer. We found that thermophilic behavior of the polymer is induced by predominantly repulsive monomer–solvent interactions, whereas thermophobic behavior is observed when attractive interactions dominate. The temperature preference of the polymer agrees well with results by Lüsebrink et al. qualitatively, where they studied colloids in a temperature gradient via computer simulation using similar potentials.^22^ Our findings further agree well with experimental research on PEO in a water/ethanol mixture, which exhibited similar thermophoretic behavior.^11,12^ By comparing simulations of polymers with different degree of polymerization, *N* = 20 and *N* = 50, we could show that the thermophoretic mobility is independent of *N*, suggesting that thermophoresis is a monomer property. Regarding the thermophoretic behavior of diblock copolymers it turned out that the thermophoretic mobility results as a linear interpolation between the two corresponding homopolymers, depending only on the fractional number of monomers in each of the two blocks.

We have considered the behavior of diblock copolymers while ignoring other possible permutations of monomer order in copolymers. Diblock copolymers represent the most polar case of bipolymers, and therefore, we would expect specific copolymer behavior to arise from the monomer arrangement in blocks the most. However, despite the conformational asymmetry that block copolymers offer, the thermophoretic motion scaled linearly with monomer number fraction. Hence, we expect all other permutations of copolymers to behave in a similar manner.

Experimental work on block copolymers in a temperature gradient showed that the thermophoretic motion is dominated by the well soluble block.^7^ The reason is that the well soluble copolymer ends form a protective outer shell for the poorly soluble ends, which leads to draining of solvent from the core and therefore diminished interaction between the polymer core and the solvent. In our simulations, we observed neither the wrapping nor the domination of the good-solvent polymer end in the thermophoretic motion. Rather, the thermophoretic motion of the copolymers behaved interpolatingly between the two ends. We propose that the reason for that is the fact that the polymers we simulated were rather short (*N* ≤ 50), and also, we only simulated single polymers in each half-box. This does not give the polymer enough phase space to form micelles and exhibit micelle behavior. It is likely, that this experimentally observed effect of a dominating outer shell behavior can be reproduced by simulations using either more instances of polymers in each half-box, such that they assemble into micelles, or simulating much longer block copolymers such that the good-solvent block is long enough to wrap around the poor solvent block, and therefore shielding it from solvent interaction.

In our simulations, we found that polymer conformation does not play any significant role for thermophoretic motion. The thermophoretic mobility of both, homopolymers with good solvent quality and with poor solvent quality, did not scale with monomer number *N* in any way, and could therefore be classified as a monomer property. However, extrapolating from our thoughts on block copolymers and the formation of micelles, for large polymers with poor solvent quality (i.e., quasi colloids with almost crystalline core) we do not expect *N*-independent thermophoretic motion anymore. This is because also here, the core is shielded from solvent–interaction by the outer layer and would therefore not contribute to the thermophoresis. We expect this effect to be visible only for polymers with *N*^1/3^ ≫ 1, as only then a significant number of monomers can be shielded by the outer layer.

## Supporting Information Available

Results for simulations at constant temperature and simulations with a temperature gradient when using σ as the variable parameter (PDF)
