# Supplementary material for: Polymer Thermophoresis by Mesoscale Simulations
Source: Macromolecules. 2024 Dec 4;57(24):11534–49. doi: 10.1021/acs.macromol.4c01656 (PMC11684348; doi:10.1021/acs.macromol.4c01656)
Supplement: Supplementary file 1 — ma4c01656_si_001.pdf [file ma4c01656_si_001.pdf]

Supporting Information: Polymer thermophoresis by mesoscale simulations

Lisa Sappl,* Christos N. Likos,* and Andreas Zöttl*

Faculty of Physics, University of Vienna, Boltzmanngasse 5, 1090 Vienna, Austria

E-mail: lisa.sappl@univie.ac.at; christos.likos@univie.ac.at; andreas.zoettl@univie.ac.at

1 Simulations using polymer-solvent interactions with σ as the free parameter

In this section, we investigate the thermophoretic behavior of polymers when using σ as a free parameter instead of λ. Specifically, we apply the following potential between monomers and solvent particles:

$$U_{\mathrm{ms}}(r) = \begin{cases} 4k_{\mathrm{B}}T_0 \left[\left(\frac{\sigma}{r}\right)^{12} - \left(\frac{\sigma}{r}\right)^{6} + \frac{1}{4}\right] & \text{if } r \leq 2^{1/6}\sigma, \\ 0 & \text{if } r > 2^{1/6}\sigma, \end{cases} \tag{1}$$

by setting $\lambda = 0$ and $n = 6$.

1.1 Simulations at constant temperature with varying σ

Varying the effective diameter σ directly controls the radius of gyration R_{G} of the polymer. The quantity σ can be viewed as a sum of the monomer radius and the solvent particle radius. For monomer-monomer interactions, we fixed $\sigma_{\mathrm{mm}} = a$, indicating that the monomer radius is $a/2$. By setting $\sigma = 0.5a$, we attribute zero excluded volume to solvent particles

regarding interactions with monomers. As a result, for the chosen parameters, the polymer adopts an expanded, good solvent state. When we increase σ, we increase the excluded volume that solvent particles exhibit with respect to monomers. This leads to monomers and solvent particles separating from each other and thus forces the polymer to adopt more compact conformations, eventually classifying as poor solvent regime for sufficiently high σ. We plot $R_{\mathrm{G}}^2/(N-1)$ against σ for polymers with different degrees of polymerization N to find the Θ point at approximately $\sigma = \sigma_\Theta \approx 0.692$, see Fig. S1. For $\sigma < \sigma_\Theta$ the polymer

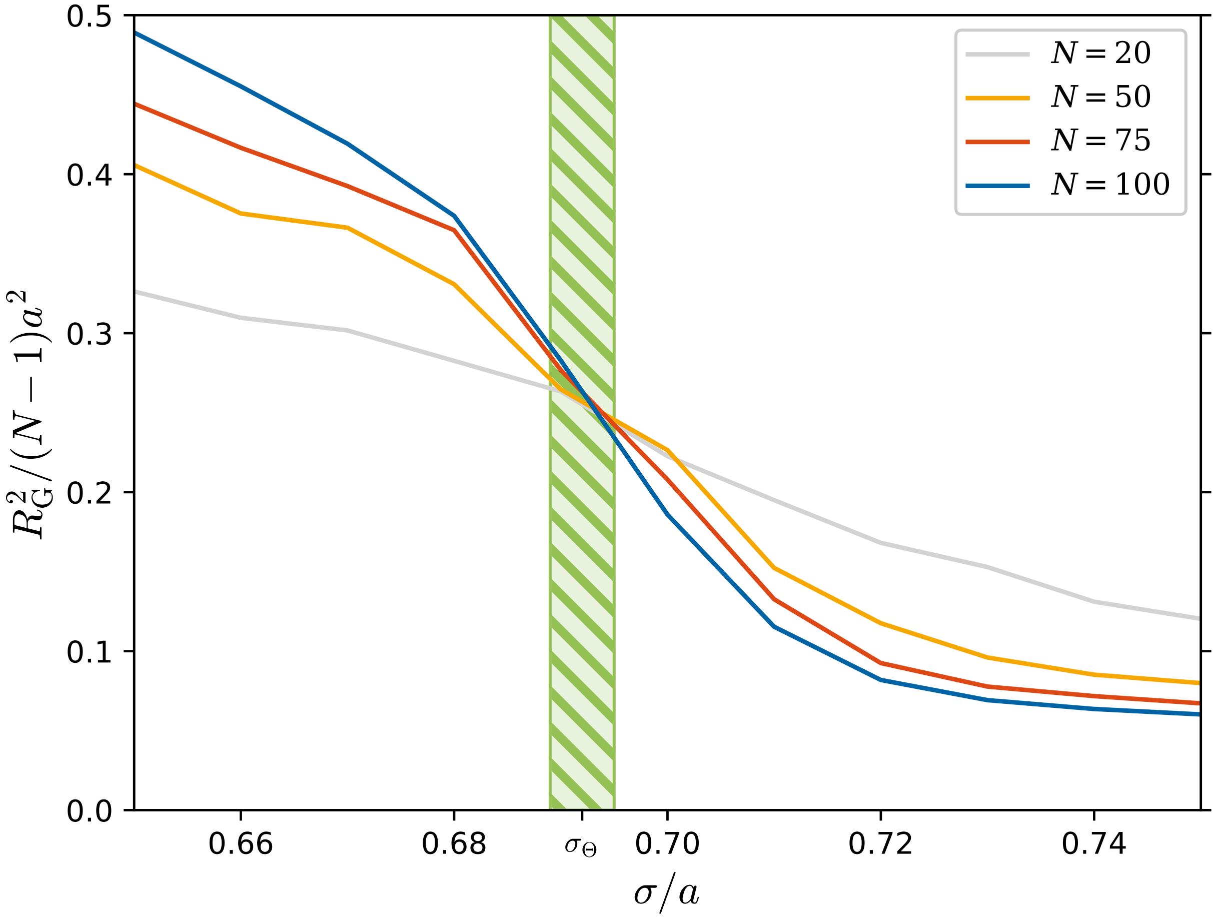

Figure S1: The Θ point when using σ as the variable parameter and keeping $\lambda = 0$ fixed. It is located at $\sigma = \sigma_\Theta \approx 0.692$. Note, for $N = 20$ (gray) the polymer is too short to expect good scaling behavior.

enters the good solvent regime, while for $\sigma > \sigma_\Theta$, we find the polymer in a poor solvent state.

1.2 Thermophoresis of homopolymers with varying σ

Fig. S2 shows the ensemble-averaged center-of-mass displacement of polymers with length $N = 20$ in a temperature gradient using the interaction potential given by Eq. 1, setting the effective diameter to $\sigma = 0.5a$, Fig. S2a and $\sigma = 0.7a$, Fig. S2b. The polymer in Fig. S2a

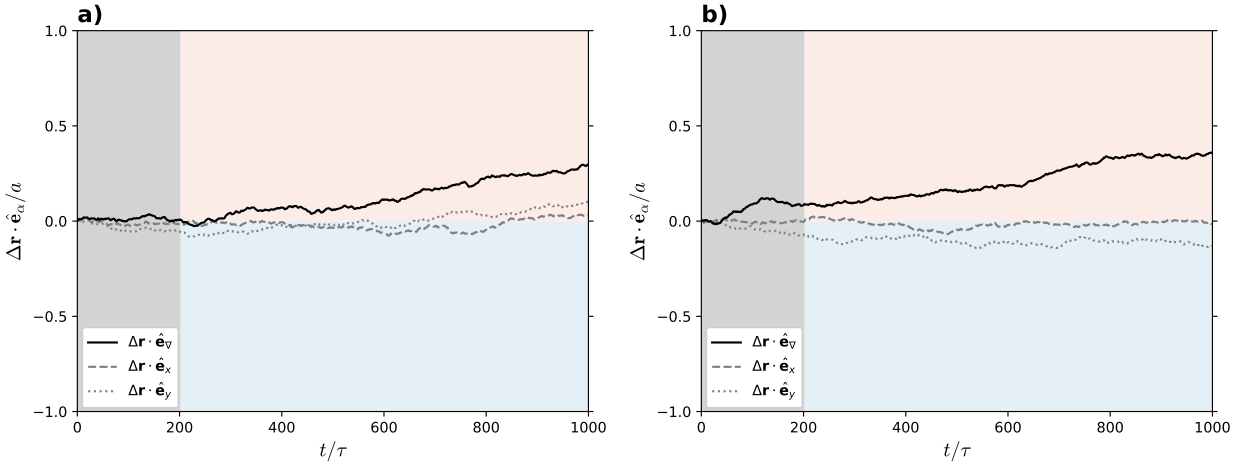

Figure S2: The ensemble-averaged displacement of the polymer center-of-mass along the box axes over time by using σ as the variable parameter in the pair-potential, shown for a) $\sigma = 0.5a$, and b) $\sigma = 0.7a$. Both show an average over 480 runs with 2 polymers of $N = 20$.

is well within the good solvent regime, whereas the polymer in Fig. S2b represents the poor solvent regime (see also Fig. S1). Nevertheless, both show distinct thermophilic behavior, which we attribute to the choice of $\lambda = 0$. Furthermore, the thermophoretic drifts of both polymers appear to be similar in magnitude. From these figures, we couldn't conclude any significant influence of σ on thermophoresis, and therefore, we decided to keep only the interaction parameter λ as our control parameter as described in the main text. Although σ has no effect on thermophoresis, it clearly has an effect on polymer size, indicating that there is a decorrelation between solvent quality and thermophoretic behavior.

2 Soret coefficient of homopolymers

Using the obtained simulation data, we can calculate the Soret coefficient

$$S_{\mathrm{T}} = \frac{D_{\mathrm{T}}}{D} \tag{2}$$

for different interaction parameters λ. The results are presented in Fig. S3. The plateau region that we observed for $D_{\mathrm{T}}(\lambda)$, here shows a tail that decreases in magnitude for $S_{\mathrm{T}}(\lambda)$. This can be attributed to the fact that for smaller λ, hence poorer solvent quality, the polymer shrinks, which enhances diffusion and therefore in turn decreases S_{T}.

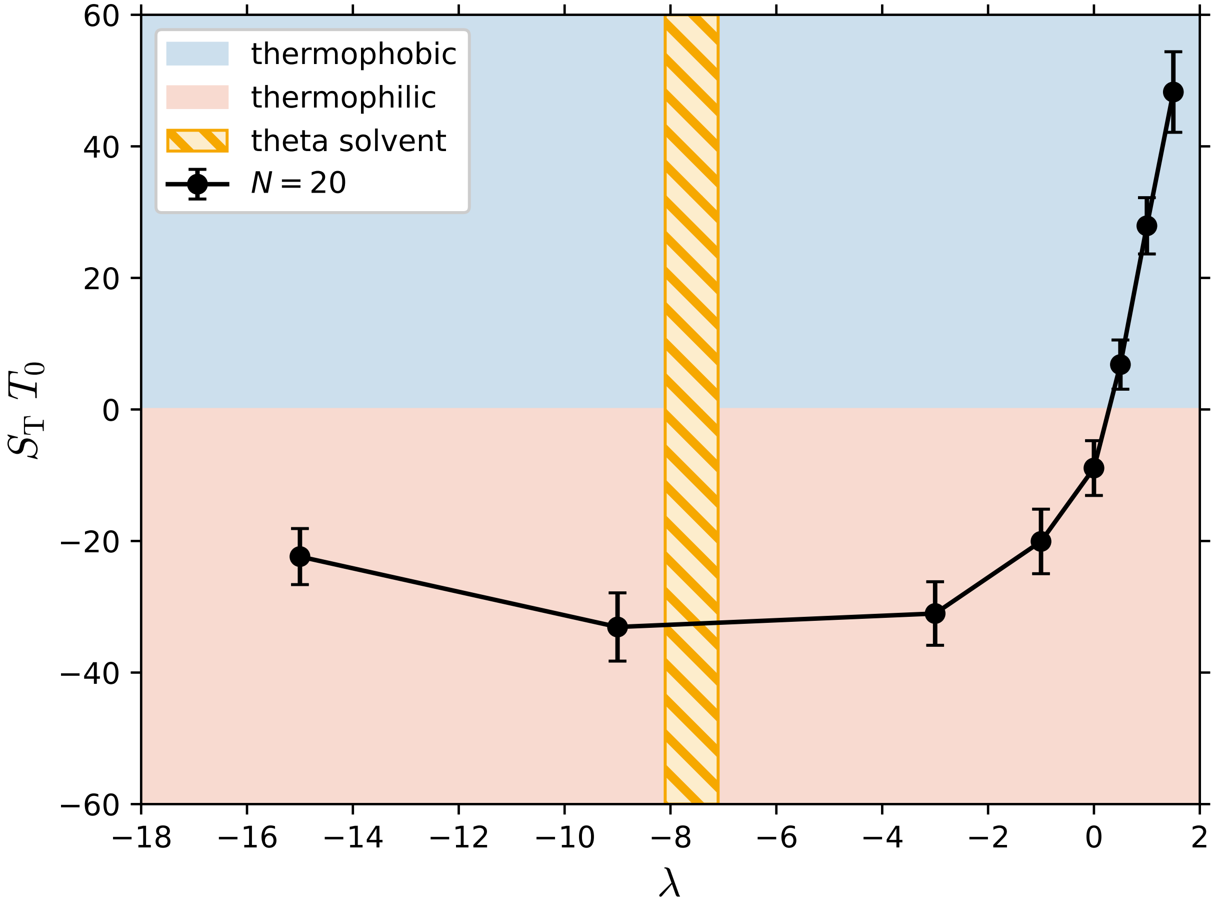

Figure S3: The finite-size Soret coefficient S_T for different interaction parameters λ of polymers with $N = 20$. Thermophilic polymer behavior is represented by the red region, thermophobic behavior is represented by the blue region. Error bars show estimates following Gauss' law of error propagation of the relative standard deviation $s(S_T)/S_T = \sqrt{(s(D_T)/D_T)^2 + (s(D)/D)^2}$, of which the standard deviations $s(D_T)$ and $s(D)$ were obtained from the standard deviations of the means in the ensemble average. The Θ point regarding solvent quality is found at $\lambda_\Theta \approx -7.6$, represented by the dashed yellow bar. $\lambda < \lambda_\Theta$ gives the poor solvent quality regime, whereas $\lambda > \lambda_\Theta$ give good solvent quality.
